# One Full or Two Fractional Doses of Inactivated Poliovirus Vaccine for Catch-up Vaccination in Older Infants: A Randomized Clinical Trial in Bangladesh^[Author-notes jiac205-FM2]^

**DOI:** 10.1093/infdis/jiac205

**Published:** 2022-05-16

**Authors:** Asma B Aziz, Harish Verma, Visalakshi Jeyaseelan, Mohammad Yunus, Samarea Nowrin, Deborah D Moore, Bernardo A Mainou, Ondrej Mach, Roland W Sutter, Khalequ Zaman

**Affiliations:** International Centre for Diarrheal Disease, Bangladesh, Dhaka, Bangladesh; International Vaccine Institute, Seoul, South Korea; World Health Organization, Geneva, Switzerland; World Health Organization, Geneva, Switzerland; International Centre for Diarrheal Disease, Bangladesh, Dhaka, Bangladesh; International Centre for Diarrheal Disease, Bangladesh, Dhaka, Bangladesh; Centers for Disease Control and Prevention, Atlanta, Georgia, USA; Centers for Disease Control and Prevention, Atlanta, Georgia, USA; World Health Organization, Geneva, Switzerland; World Health Organization, Geneva, Switzerland; International Centre for Diarrheal Disease, Bangladesh, Dhaka, Bangladesh

**Keywords:** inactivated poliovirus vaccine, fractional IPV, polio, Bangladesh, older cohort

## Abstract

**Background:**

The polio eradication endgame called for the removal of trivalent oral poliovirus vaccine (OPV) and introduction of bivalent (types 1 and 3) OPV and inactivated poliovirus vaccine (IPV). However, supply shortages have delayed IPV administration to tens of millions of infants, and immunogenicity data are currently lacking to guide catch-up vaccination policies.

**Methods:**

We conducted an open-label randomized clinical trial assessing 2 interventions, full or fractional-dose IPV (fIPV, one-fifth of IPV), administered at age 9–13 months with a second dose given 2 months later. Serum was collected at days 0, 60, 67, and 90 to assess seroconversion, priming, and antibody titer. None received IPV or poliovirus type 2-containing vaccines before enrolment.

**Results:**

A single fIPV dose at age 9–13 months yielded 75% (95% confidence interval [CI], 6%–82%) seroconversion against type 2, whereas 2 fIPV doses resulted in 100% seroconversion compared with 94% (95% CI, 89%–97%) after a single full dose (*P* < .001). Two doses of IPV resulted in 100% seroconversion.

**Conclusions:**

Our study confirmed increased IPV immunogenicity when administered at an older age, likely due to reduced interference from maternally derived antibodies. Either 1 full dose of IPV or 2 doses of fIPV could be used to vaccinate missed cohorts, 2 fIPV doses being antigen sparing and more immunogenic.

**Clinical Trial Registration:**

NCT03890497.

## Teaser

IPV immunogenicity increased when administered at an older age, likely due to reduced interference from maternally derived antibodies. Two doses of fIPV could also be used to vaccinate missed cohorts, which is antigen sparing and more immunogenic.

The Global Polio Eradication Initiative (GPEI) has been successful in decreasing the number of wild poliovirus (WPV)-endemic countries from >125 in 1988 to 2 in 2021 and the number of cases from >350 000 to 2 [1]. Since 1988, an estimated 20 million children did not acquire paralytic poliomyelitis (the “crippling consequences of poliovirus infection”) due to polio eradication efforts [2]. Of the 3 serotypes of WPV (types 1, 2, and 3), types 2 and 3 were certified eradicated in 2015 [3] and 2019 [4–6], respectively. Afghanistan and Pakistan are the remaining WPV type 1 endemic countries [7–9]. Despite the progress, the goal of global eradication remains elusive.

Polio eradication requires the removal of all polioviruses from populations [5], including WPVs and vaccine-derived polioviruses (VDPVs). VDPVs are derived from the Sabin viruses in the oral poliovirus vaccine (OPV). VDPVs may emerge after prolonged replication in OPV recipients or protracted circulation in undervaccinated populations. Because the viruses may then establish endemic or epidemic transmission, the continued use of OPV after eradication is not compatible with eradication [6]. To resolve the VDPV issue, the GPEI polio eradication endgame plans require the removal of OPV. As a first step, Sabin type 2 poliovirus was removed from trivalent OPV in 2016 and replaced by bivalent (types 1 and 3) OPV (bOPV) [7]. At the same time, ≥1 dose of inactivated poliovirus vaccine (IPV) was introduced in routine immunization programs for risk mitigation purposes [8].

However, IPV supplies were insufficient to meet global demands until 2019 [9], requiring some countries to delay introduction or apply mitigation measures. Some countries stretched available IPV supplies by introducing 2 fractional-dose IPV (fIPV; 0.1 mL given intradermally) as recommended by the World Health Organization (WHO) [10]. This strategy with 2 fIPV doses required 60% less antigen than a single full dose (0.5 mL given intramuscularly). Unfortunately, nearly 42 million infants did not receive any doses of IPV because of supply constraints from April 2016 to 2018 [11].

Bangladesh was one of the countries affected by IPV shortages. It was a lower priority for IPV supplies because no WPV cases had been detected after 2006, having achieved high routine immunization coverage of >90% [12] and having been officially certified polio free in 2014 [13]. No IPV was available in Bangladesh from April 2016 to November 2017 [14]. Therefore, a large cohort of children born during this period did not receive poliovirus type 2-containing vaccine. In November 2017, the country introduced 2 fIPV doses at 6 and 14 weeks in the immunization schedule.

A substantial body of evidence demonstrates that the immunogenicity of 1 full dose IPV is superior to that of fIPV, and 2 fIPV doses are noninferior to 2 full doses of IPV among young infants [15–19]. However, no study had compared the immunogenicity of fIPV or IPV in older infants. Hence, the primary focus of this study was to generate data on type 2 immunogenicity of full or fractional doses of IPV in 9 to 13-month-old infants who had received bOPV in the primary schedule but were naive to poliovirus type 2 vaccines.

## METHODS

### Study Design

**Figure 1.**
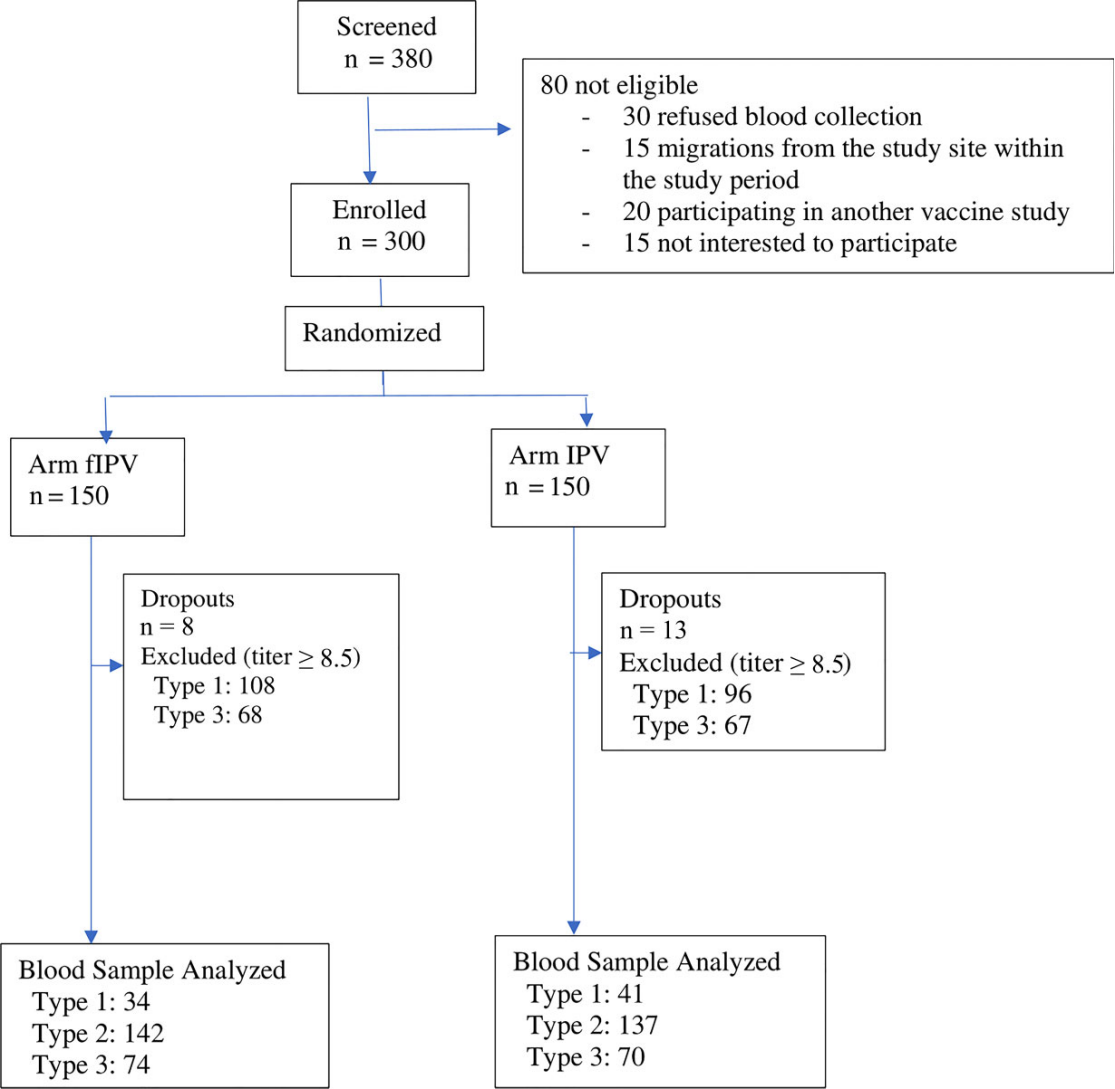
Consort flowchart. Abbreviations: fIPV, fractional-dose IPV; IPV, inactivated poliovirus vaccine.

This study was an open-label randomized trial with 2 groups of intervention. The study was conducted between September 2018 and September 2019 in the International Centre for Diarrheal Disease, Bangladesh (icddr, b) rural Matlab intervention area (blocks A, B, C, D) and Mirpur, urban slum (section 1–14) in Dhaka, Bangladesh. Healthy children (as assessed by a study physician) who were 9–13 months of age and who had not received IPV were eligible for the study. After meeting all enrollment criteria and providing informed consent, participants were randomly assigned to receive a first dose of fIPV or full-dose IPV and a second dose after 2 months. The study design is shown in the consort flowchart (Figure 1).

The study was reviewed and approved by the Research Review Committee and Ethical Review Committee of the icddr, b, and the Ethical Review Committee, WHO, Geneva, Switzerland. The study was conducted in compliance with Good Clinical Practice guidelines and registered with ClinicalTrials.gov, NCT03890497.

### Randomization and Masking

Enrolled children were randomly allocated to 1 of the 2 groups using permuted block randomization of varying proportions (block sizes of 2, 4, and 6), generated using R. The parent or the study investigator had no discretion to opt for a particular study group. This study was open label because the dose and route of administration were different between the groups. However, laboratory investigators at the Centers for Disease Control and Prevention (CDC), Atlanta, GA, assessed the outcome blinded to the vaccine allocation.

### Study Procedures

Study investigators (A.B.A., S.N., K.Z.) in Matlab and Mirpur listed children born after April 2016 and checked immunization history recorded in the immunization records and the health cards for the Matlab site. Study information was shared with parents whose children had not received IPV and, if interested, they were invited to participate. Upon arrival at the medical center, registration was done, and the child’s weight and height were recorded. Children whose parents could understand and comply with planned study procedures, including not moving outside the study area during the study period, who provided written consent for their child’s participation, were then enrolled and randomized to 1 of the 2 study groups as per the study design.

A venous blood sample (2 mL) was collected under sterile conditions from each participant. Once the blood was collected, the participant was administered the study vaccine by a trained nurse taking all necessary aseptic precautions. fIPV 0.1 mL was administered intradermally at the upper-third of the deltoid region of the right arm using a 0.1-mL autodisable syringe as per national immunization guidelines. IPV 0.5-mL dose was administered intramuscularly at the anterolateral aspect of the upper right thigh with an autodisable syringe. A questionnaire was filled with all the necessary information. The second study visit was scheduled 2 months after enrollment. A blood sample was collected during this second visit, and a second vaccine was administered. Blood samples were also collected during the subsequent visits (1 week and 1 month after receiving the second dose of the study vaccine). The study physician asked parents about AEs, and vaccinations received since the last visit at each visit.

Blood samples (2 mL) collected from the study sites were transported to the icddr, b laboratory at 2–8°C. Within 24 hours of collection, serum was separated by centrifugation and stored at −20°C until the last visit of the last subject. Then all sera were shipped to the CDC laboratory in Atlanta, GA, for testing antibody titers to the 3 poliovirus serotypes using a microneutralization assay [20].

Participants could be withdrawn from the trial if parents withdrew consent, or the participant was lost to follow-up or received another investigational vaccine during the study. Study participants could also withdraw for any reason at any time and were not replaced. The reason for withdrawal was recorded.

### Study Vaccine

IPV produced by Bilthoven Biologicals, Bilthoven, the Netherlands, was used in the trial. The IPV contained 40, 8, and 32 D-antigen units against poliovirus types 1, 2, and 3, respectively, and was stored in a refrigerator at the icddr, b facilities at 2–8°C with 24-hour generator backup. Separate multidose vials were used for IPV and fIPV groups and carried to the study clinic in a cold box with a temperature monitoring device. WHO multidose open-vial policy was strictly followed, and vial opening date and time were put on the label. From each vial of 2.5 mL assigned for fIPV use (equivalent to 25 doses), 20 participants received the vaccine dose of 0.1 mL each. From each vial (2.5 mL, equivalent to 5 full doses) assigned for a full dose, 4 participants were vaccinated with 0.5 mL each. Each vial was used within 28 days of the opening, and the vaccine vial monitor was checked each time before vaccination. The used labeled vials with the residual vaccine were stored in the cold chain as a backup for vaccine accountability or potential potency testing.

### Adverse Events

AEs were defined as any illness occurring in participants during the study period. Serious adverse events (SAEs) were defined as death, life-threatening events, hospital admission or prolongation of existing hospitalization, paralysis or severe disability, and anaphylactic reaction after vaccine administration. Immediate AEs were captured through observation for 30 minutes after vaccination. In addition, home visits were done within 48 hours after administration of the first and second dose of vaccine to collect systemic and injection-site AEs data. At each subsequent study visit, the physician enquired about any events between the visits and performed a physical examination to detect and record any events. Parents were instructed to seek care immediately and contact the study clinics if their child became ill between the scheduled study visits. SAEs were captured throughout the study period. The principal investigator reviewed all AE reports, and all SAE reports were shared within 24 hours with the icddr, b’s Institutional Review Board, the Data Safety and Monitoring Board, and WHO.

### Outcome Assessment

We used the following definitions: (1) seroconversion after 1 and 2 doses of IPV or fIPV, for seronegative participants (reciprocal titer <8) at enrollment, a change to seropositivity in a successive specimen (ie, a reciprocal titer of ≥8) indicated seroconversion; (2) seropositivity was defined as reciprocal antibody titer ≥8; (3) for participants who were positive against serotypes 1 and 3 at enrollment, we used a 4-fold rise in antibody titer to indicate a boosting immune response (because all participants had a history of 3 doses of bOPV prior to study participation); to calculate a 4-fold rise, we had to exclude participants with a reciprocal antibody titer of >362; (4) for participants who were positive against serotype 2 at enrollment, we also considered a 4-fold rise over expected antibody decline to indicate seroconversion because these participants had not received any type 2-containing vaccine; (5) priming was defined as 4-fold rise of the antibody titer 7 days after a second dose of vaccine in participants who remained seronegative after the first dose [17]; and (6) reported median titers were restricted to those seropositive participants, except the enrollment titers that were for all participants, as this provides a better indication of vaccine performance.

### Sample Size

Accepting precision of ±8% with 95% confidence, we calculated that a minimum of 140 participants in each of the 2 study groups would be needed. This sample size is based on a scenario of 63% immunogenicity after 1 IPV dose [17]. We increased the sample size to 150 per group to account for attrition.

### Statistical Analysis

Statistical analyses were performed with STATA [21]. Descriptive analyses were presented for baseline characteristics and AEs in the 2 groups. Median titers with 95% bootstrap confidence intervals were calculated in each group. In this article, we report the modified per-protocol analyses. We used χ^2^ tests to compare all proportions in baseline attributes (where appropriate) and seroconversion rates.

## RESULTS

### Study Population

Between 28 September 2018 and 26 June 2019, parents of 380 potentially eligible children were approached for study participation, and 300 children fulfilling eligibility criteria were enrolled. These participants were randomly allocated to 1 of the 2 groups. A total of 279 participants (93%) completed the study (Figure 1). Among the 21 participants who did not complete the study, 8 were in the fractional-dose group and 13 were in the IPV full-dose group, and the reasons were as follows: 13 parents withdrew consent and 8 participants were lost to follow-up. None of those children whose parents withdrew consent to continue the study were due to suspected or confirmed AEs following immunization.

The baseline demographic characteristics of the 2 study groups were comparable at enrollment. Poliovirus seroprevalence in the fIPV and IPV arms were 83% and 84% for type 1, 9%, and 11% for type 2, and 75% and 81% for type 3, respectively, and median titers were similar between the 2 groups for all 3 serotypes (Table 1).

**Table 1.** Baseline Attributes—Demographics and Seroprevalence

Variables	fIPV (n = 150)	IPV (n = 150)
Median age, mo (IQR)	11 (10–12)	11 (9–12)
Male sex, No. (%)	77 (51.3)	72 (48)
Mother’s education, No. (%)		
No formal school	41 (27.3)	41 (27.3)
Primary	41 (27.3)	38 (25.3)
Middle	47 (31.3)	53 (35.3)
High	19 (12.7)	16 (10.7)
Graduate	2 (1.3)	2 (1.3)
Poliovirus type 1		
Seroprevalence, No. (%)	125 (83.3)	126 (84.0)
95% CI of proportion	76.6–88.4	77.3–89.0
Median titer (95% CI)^a^	≥1448 (910 to ≥1448)	1300 (910 to ≥1448)
Poliovirus type 2		
Seroprevalence, No. (%)	13 (8.7)	16 (11.3)
95% CI of proportion	5.1–14.3	7.2–17.4
Median titer (95% CI)^a^	<8 (<8 to <8)	<8 (<8 to <8)
Poliovirus type 3		
Seroprevalence, No. (%)	112 (74.7)	122 (81.3)
95% CI of proportion	67.2–81.0	74.3–86.8
Median titer (95% CI)^a^	288 (144–455)	325 (181–455)

No significant differences were detected between the study groups.

Abbreviations: CI, confidence interval; fIPV, fractional-dose IPV; IPV, inactivated poliovirus vaccine; IQR, interquartile range.

a 95% Bootstrap confidence interval.

### Immunogenicity

#### Poliovirus Type 2

A total of 107/142 (75%) participants in the fractional-dose group and 129/137 (94%) participants in the full-dose group (*P* < .001) seroconverted after the first dose of vaccine. The priming immune response (7 days after a second vaccine dose) was 35/35 (100%) in the fIPV and 6/6 (100%) in the IPV arms. The cumulative 2-dose seroconversion was 100% in the fractional-dose group and 100% in the full-dose group (Table 2). Two fractional doses resulted in a 100% seroconversion compared with 94% after a single full dose (*P* < .001). The median reciprocal antibody titers against poliovirus type 2 were lower in the fractional dose compared to the full-dose arm after receiving the first dose at 9–13 months (16), increased 1 week after administration of the second fractional dose at 11–15 months of age, and then again declined rapidly 3 weeks later (Table 2).

**Table 2.** Rates of Seroconversion and Priming Immune Response after 1 or 2 Doses of Inactivated Poliovirus Vaccine for Poliovirus Types 1, 2, and 3

Poliovirus Type	fIPV		IPV		*P* Value
	n/N	SC, % (95% CI) Median Titer (95% CI^a^)	n/N	SC, % (95% CI) Median Titer (95% CI^a^)	
After the first dose					
Type 1	18/34	53 (36.7–68.5)	34/41	83 (69–91)	.005^b^
		≥1448 (≥1448 to ≥1448)		≥1448 (≥1448 to ≥1448)	.7
Type 2	107/142	75 (68–82)	129/137	94 (89–97)	<.001^b^
		36 (18–45)		144 (91–258)	<.001^b^
Type 3	52/74	70 (59–79)	55/70	79 (68–87)	.254
		≥1448 (≥1448 to ≥1448)		≥1448 (≥1448 to ≥1448)	.167
Priming					
Type 2	35/35	100	6/6	100	.525
		1152 (910–1152)		≥1448 (≥1448 to ≥1448)	<.001^b^
Cumulative after 2 doses					
Type 1	34/34	100	41/41	100	
		≥1448 (≥1448 to ≥1448)		≥1448 (≥1448 to ≥1448)	.466
Type 2	142/142	100	137/137	100	
		455 (362–724)		≥1448 (1152 to ≥1448)	<.001^b^
Type 3	73/74	99 (93–100)	70/70	100	
		≥1448 (≥1448 to ≥1448)		≥1448 (≥1448 to ≥1448)	.407

N, participants who were baseline sero-negative (reciprocal titer <8); n, number of participants who seroconverted after first dose or second dose (cumulative). For priming: N, participants who did not seroconvert after first dose; n, Participants seroconverted after 1 week of second dose.

Abbreviations: CI, confidence interval; fIPV, fractional-dose IPV; IPV, inactivated poliovirus vaccine; SC, seroconversion.

a 95% Bootstrap CI.

b Significant, *P* value < .05.

#### Poliovirus Type 1

A total of 18/34 (53%) participants had an immune response in the fIPV arm versus 34/81 (83%) participants in the IPV arm after a first dose, and 10/10 (100%) and 19/19 (100%) had a booster response. After a second dose, 16/16 (100%) and 7/7 (100%) seroconverted to fIPV or IPV, respectively. The cumulative 2-dose immune response (seroconversion and boosting) was 34/34 (100%) to fIPV and 41/41 (100%) to IPV. After the first dose of vaccine, the median reciprocal antibody titers against poliovirus type 1 were higher in the full-dose arm, similar 7 days after the second dose, and remained stable 3 weeks later (Table 2).

#### Poliovirus Type 3

A total of 52/74 (70%) and 55/70 (79%) had an immune response following a dose of fIPV or IPV, respectively; 37/38 (97%) after fIPV and 44/44 (100%) after IPV had a booster response. After a second dose, 20/21 (95.2%) and 15/15 (100%) seroconverted, and 4/53 (8%) and 6/55 (11%) had a booster response. The cumulative 2-dose response was 73/74 (99%) and 70/70 (100%) for fIPV and IPV, respectively. The median reciprocal antibody titers against poliovirus type 3 increased after 1 dose in both groups and remained the same after the second dose (Table 2).

### Adverse Events

A total of 65 AEs were reported. Only 1 (1%) of these were classified as SAE. A participant in the fIPV group was hospitalized due to enteric fever and wholly recovered after treatment; none of the AEs were attributed to poliovirus vaccines. The most commonly reported AEs were fever with the common cold (11), acute watery diarrhea (11), acute respiratory infections (10), and common cold (9). There were 6 events with dermatological conditions (scabies and tinea capitis). Other reported events included fever (4), conjunctivitis (4), febrile convulsions (2), pneumonia (2), and single reports of insect bite, cough, acute otitis media, dysentery, and vomiting.

## DISCUSSION

This is the first study to provide immunogenicity data (focusing on serotype 2) on IPV vaccination among 9 to 13-month-old infants naive to poliovirus type 2. Our study demonstrated (1) higher than expected seroconversion rates following a single dose of either fIPV or IPV; (2) almost universal immunity (seroconversion and antibody titers after the 2-dose schedule of either fIPV and IPV); (3) 100% of vaccinees responded with a priming immune response after the first dose; and most importantly (4) 2 fIPV doses resulted in significantly higher seroconversion rates than a single full dose of IPV.

In the 1950s and 1960s, the potency of IPV was calibrated to overcome maternally derived antibodies induced by WPV infection [22]. A large body of scientific evidence supports the efficacy of IPV or fIPV in infants aged ≤6 months [15, 18]. However, maternally derived antibodies wane with increasing age. Therefore, later administration of the first dose of IPV should increase its immunogenicity [23–26]. While the early administration of IPV in 3-dose schedules results in >80% seroconversion against all 3 serotypes [26, 27], a single dose of IPV given in our study at 9–13 months of age seroconverted 94% of participants. Similarly, 2 doses of fIPV resulted in 100% seroconversion. A study from Cuba with a first dose at 4 months but a longer interval of 4 months to the second dose reported seroconversion rates of 98% and 100% with 2 doses of fIPV and 2 doses of IPV, respectively, demonstrating that both age of administration of the first dose and the interval between doses continue to be essential factors for IPV response [17]. An earlier study in Bangladesh reported a 47% seroconversion rate after 1 IPV dose was administered at 14 weeks against poliovirus type 2 [15]. In addition, 2 IPV doses from another study also in Bangladesh showed 91% seroconversion [16], and 2 fIPV doses administered at 6 and 14 weeks seroconverted 64% [15] and 81% [16], respectively, against poliovirus type 2.

In our study, all participants responded with a priming immune response after the first dose of fIPV or IPV, suggesting that the first-dose immunity was sufficiently robust to respond with an anamnestic response following a second dose. These data indicate that a similar rapid anamnestic response could be expected following exposure to a circulating poliovirus.

Our study also provided information on closing the immunity gaps to poliovirus types 1 and 3. Because all participants had a history of receiving 3 doses of the bOPV vaccine, only a small proportion was seronegative at study enrollment. The single-dose seroconversion rates were 33% and 68% to poliovirus type 1 and similar (42%) to poliovirus type 3 after fIPV or IPV (Supplementary Tables). This was much lower than that observed in type-2-naive participants, suggesting that the preexisting immunity interfered with seroconversion. However, the immunity gaps were almost completely closed after 2 doses of fIPV or IPV. We also noted a significantly higher seroconversion after IPV for type 1 but not for type 3, for reasons not entirely apparent.

Our study had limitations. The study was designed to guide catch-up vaccination with IPV in type-2-naive older infants and children. Therefore, our results cannot be generalized to types 1 and 3 (because of their 3-dose bOPV history). To use a boosting immune response definition, we had to exclude large numbers of participants with high antibody titers to poliovirus types 1 (n = 204) and type 3 (n = 135), limiting the power of the related analyses. For poliovirus type 2, 30/300 (10%) participants were seropositive at enrollment. This low baseline type 2 seropositivity could be attributed to the persistence of maternally derived maternal antibodies, low-grade undetected community transmission of VDPV2, or the inadvertent receipt of a type-2-containing vaccine dose outside the study. However, IPV was not available in Bangladesh’s public or private sector. Therefore, we assumed that the seropositive participants had persistent maternally derived antibodies and used the standard seroconversion criteria (4-fold rise over expected decline) for naive infants. If we were to use the more conservative 4-fold rise definition, only a single subject in the fIPV arm would have to be reclassified as a nonresponder. Practically, this would not change any findings or interpretation. In conclusion, 1 full dose of IPV or 2 fIPV doses are almost equivalent in inducing immunity against poliovirus type 2. However, the 2-dose fIPV schedule offers some immunological advantages, including significantly higher seroconversion rates and meeting the prime-boost model, resulting in a 3-fold increase in antibodies. Interestingly, 2 fIPV doses close the immunity gaps to poliovirus types 1 and 3, whereas 1 full IPV dose does not. Programmatically, a 2-dose fIPV is also dose sparing (and therefore cost sparing), requiring only 40% of antigen compared to 1 full dose of IPV, but requires 1 additional health center visit. On balance, it seems that a 2-dose fIPV schedule offers the best balance of immunity and cost for catch-up vaccination of missed cohorts of older infants and children. fIPV has been adopted in routine immunization schedules in 6 countries with almost 30% of the global birth cohort [28], offering substantial economic gains by reducing massive polio vaccine wastage and better immunity. But still some countries are reluctant to adopt fIPV as this is considered off-label use. Countries without experience using fIPV should carefully conduct risk-benefit analysis from a programmatic perspective considering critical points, including IPV supply, training of vaccinators, and proper advocacy and communication.

## Supplementary Data

Supplementary materials are available at *The Journal of Infectious Diseases* online (http://jid.oxfordjournals.org/). Supplementary materials consist of data provided by the author that are published to benefit the reader. The posted materials are not copyedited. The contents of all supplementary data are the sole responsibility of the authors. Questions or messages regarding errors should be addressed to the author.

## Supplementary Material

jiac205_Supplementary_Data

## Notes

***Acknowledgments***. The International Centre for Diarrheal Disease, Bangladesh (icddr, b) acknowledges with gratitude the World Health Organization’s (WHO’s) commitment to the research support. icddr, b is also grateful to the Governments of Bangladesh, Canada, Sweden, and the United Kingdom for providing core and unrestricted support. The authors acknowledge the efforts and contributions of the entire study team at the study sites and the community level staff of Matlab and Mirpur catchment areas. We also appreciate the contributions of Bangladesh and the Ministry of Health to allow this trial and provide IPV vaccines for use in the trial. We are thankful to the team at the CDC Population Immunity Laboratory (William Hendley, Kathryn Jones, Sharla McDonald, Mario Nicholas, and Yiting Zhang) who performed neutralization assays on the serum samples. WHO Bangladesh was very supportive of this work.

***Financial support***. This work was supported by Rotary International through the WHO (grant number PP18BMF0002). Funding to pay the Open Access publication charges for this article was provided by Rotary International through the WHO (grant number PP18BMF0002).
