# Supplementary material for: One Full or Two Fractional Doses of Inactivated Poliovirus Vaccine for Catch-up Vaccination in Older Infants: A Randomized Clinical Trial in Bangladesh
Source: J Infect Dis. 2022 May 16;226(8):1319–26. doi: 10.1093/infdis/jiac205 (PMC9574668; doi:10.1093/infdis/jiac205)
Supplement: jiac205_Supplementary_Data [file jiac205_supplementary_data.docx]

**Supplementary Table 1: Seroconversion and Boosting Immune Response after One or Two Doses of Inactivated Poliovirus Vaccine for Poliovirus Types 1**

| Outcome | **Arm A (fIPV)** | | **Arm B (IPV)** | | **95% CI of the difference** | **P-value** |
| --- | --- | --- | --- | --- | --- | --- |
|  | n/N | % | n/N | % |  |  |
| Seroconversion after the first dose | 8/24 | 33.3  (18.0-53.3) | 15/22 | 68.2  (47.3-83.6) | -34.9 (-62.0- -7.8) | 0.038 |
| Boosting after the first dose | 10/10 | 100.0 | 19/19 | 100.0 |  |  |
| Seroconversion after the second dose | 16/16 | 100.0 | 7/7 | 100.0 |  |  |
| Boosting after the second dose | 3/18 | 16.7  (5.8-39.2) | 11/34 | 32.4  (19.1-49.2) | -15.7 (-39.0-7.6) | 0.329 |
| Immune response after 2nd dose | 19/34 | 55.9  (39.5-71.1) | 18/41 | 43.9  (29.9-59.0) | 12 (-10.6-34.6) | 0.357 |

**Supplementary Table 2: Seroconversion and Boosting Immune Response after One or Two Doses of Inactivated Poliovirus Vaccine for Poliovirus Types 3**

| Outcome | **Arm fIPV** | | **Arm IPV** | | **95% CI of the**  **difference** | **P-value** |
| --- | --- | --- | --- | --- | --- | --- |
|  | n/N | % | n/N | % |  |  |
| Seroconversion after the first dose | 15/36 | 41.7  (27.1-57.8) | 11/26 | 42.3  (25.5-61.1) | -0.6 (-25.5-24.3) | 1.000 |
| Boosting after the first dose | 37/38 | 97.4  (86.5-99.5) | 44/44 | 100.0 | -2.6 (-7.6-2.5) | 1.000 |
| Seroconversion after the second dose | 20/21 | 95.2  (77.3-99.2) | 15/15 | 100.0 | -4.8 (-13.9-4.3) | 1.000 |
| Boosting after the second dose | 4/53 | 7.5  (3.0-17.9) | 6/55 | 10.9  (5.1-21.8) | -3.4 (-14.3-7.5) | 0.742 |
| Immune response after 2nd dose | 24/74 | 32.4  (22.9-43.7) | 21/70 | 30.0  (20.5-41.5) | 2.4 (-12.7-17.5) | 0.858 |
